# Human Leukocyte Antigen-G (HLA-G) Polymorphism and Expression in Breast Cancer Patients

**DOI:** 10.1371/journal.pone.0098284

**Published:** 2014-05-28

**Authors:** Seri Jeong, Seho Park, Byeong-Woo Park, Younhee Park, Oh-Joong Kwon, Hyon-Suk Kim

**Affiliations:** 1 Department of Laboratory Medicine, Severance Hospital, Yonsei University College of Medicine, Seoul, Republic of Korea; 2 Department of Surgery, Severance Hospital, Yonsei University College of Medicine, Seoul, Republic of Korea; 3 Department of Laboratory Medicine, Kwandong University College of Medicine, Goyang, Republic of Korea; 4 College of Animal Bioscience & Technology, Konkuk University, Seoul, Republic of Korea; University of Alabama at Birmingham, United States of America

## Abstract

Human leukocyte antigen-G (HLA-G) is known to be implicated in a tumor-driven immune escape mechanism in malignancies. The purpose of this study was to investigate HLA-G polymorphism and expression in breast cancer. *HLA*-*G* alleles were determined by direct DNA sequencing procedures from blood samples of 80 breast cancer patients and 80 healthy controls. Soluble HLA-G (sHLA-G) was measured by enzyme-linked immunosorbent assay (ELISA) from serum specimens. HLA-G expression in breast cancer lesions was also analyzed by immunohistochemistry staining. The presence of *HLA*-*G* 3′ untranslated region (UTR) 14-bp sequence was analyzed and found to be associated with reduced risk of breast cancer susceptibility based on HLA-G expression in tissues (*P* = 0.0407). Levels of sHLA-G were higher in the breast cancer group (median 117.2 U/mL) compared to the control group (median 10.1 U/mL, *P*<0.001). The area under the receiver operating characteristic curve (AU-ROC) values of sHLA-G for differentiating breast cancer from normal controls and for detecting metastasis from other stages of breast cancer were 0.89 and 0.79, respectively. HLA-G polymorphism and expression may be involved in breast carcinogenesis and sHLA-G concentrations could be used as a diagnostic marker for detecting breast cancer.

## Introduction

Breast cancer is the most common cancer among women worldwide; approximately 1.2 million cases of breast cancer are diagnosed annually. Breast cancer is also the most frequent cause of cancer-related mortality, accounting for almost 411,000 deaths every year [1], [2]. Although the overall incidence of breast cancer has increased, early diagnosis and treatment through screening have helped reduce the mortality from breast cancer [3]. Therefore, the investigation of appropriate tumor markers for early detection and monitoring is necessary.

Human leukocyte antigen-G (HLA-G) belongs to the non-classical HLA class I family of genes and is located at chromosome 6p21 [4]. The diversity of the promoter and the 3′-untranslated region (UTR) of the *HLA-G* gene controls the expression of HLA-G protein [5]. The *HLA-G* gene encodes seven isoforms by alternative splicing of primary transcripts, which include four membrane-bound (HLA-G1, -G2, -G3, and -G4) and three soluble isoforms (HLA-G5, -G6, and -G7) [6]. Aberrant HLA-G expression has been closely associated with several pathological conditions, including transplantation, autoimmune and inflammatory diseases, viral infections, and malignancies [7]. HLA-G exerts its function mainly against natural killer (NK) cells, T lymphocytes, and antigen-presenting cells through binding to the inhibitory receptors such as immunoglobulin-like transcripts (ILT)-2/CD85j, ILT-4/CD85d, and killer cell immunoglobulin receptor (KIR) 2DL4/CD158d [8]–[10]. Furthermore, soluble HLA-G (sHLA-G) is known to interact with CD8 co-receptor and induces the apoptosis of NK and T cells [11]. Thus, increased expression of HLA-G can lead to immune tolerance of the host. The tolerogenic properties of HLA-G have beneficial effects in pregnancy, transplantation, and inflammatory diseases by reducing immune reaction responses, whereas they are deleterious in cancer and viral infections by permitting escape of tumor or virus-infected cells from anti-tumor or anti-viral responses, respectively [4].

The *HLA-G* alleles have relatively restricted polymorphism and low sequence variations in different populations [12]–[16]. Forty-seven alleles have been assigned to the *HLA-G* gene, primarily in exons 2, 3, and 4. The *HLA-G* gene also has the presence or absence of 14-base pair (bp) at the 3′-UTR [14], [17]. These *HLA-G* polymorphisms have been associated with several types of malignancies such as hepatocellular carcinoma, esophageal cancer, transitional cell carcinoma of bladder, uterine cervical cancer, and childhood neuroblastoma [18]–[22]. However, *HLA-G* genotypes in breast cancer patients have not yet been widely reported.

As one of the expressive forms of the *HLA-G* gene, sHLA-G antigens are derived from the release of membrane-bound HLA-G isoforms (HLA-G1s for HLA-G1 shedding) and from the secretion of sHLA-G isoforms themselves (HLA-G5). sHLA-G could affect anti-tumor immune reactions both locally at the tumor site and systemically through the circulation [23]. Previous studies reported that sHLA-G concentrations are significantly increased in patients with tumors such as cervical, colorectal, gastric, esophageal, and lung cancer, as well as breast cancer [7], [24], [25].

HLA-G antigens in tissue can be observed directly by immunohistochemistry. Several studies revealed that HLA-G was more frequently observed in advanced stages of the disease and tumor grade in breast cancer, [26], [27] indicating its considerable clinical relevance to breast cancer.

The aim of this study was to evaluate the diagnostic utility of sHLA-G as a tumor marker in comparison to the conventionally used cancer antigen (CA) 15-3 and carcinoembryonic antigen (CEA) markers in breast cancer. In addition, the association between HLA-G polymorphism and expression was analyzed in the same patient population to investigate the possible mechanism of HLA-G in breast carcinoma.

## Materials and methods

### 1. Ethics statement

This study was approved by the independent Institutional Review Board of Severance Hospital and informed consents were written.

### 2.Study subjects

A total of 80 patients with breast cancer, who agreed to participate in our study, signed the informed consent form and received operations at our hospital, were randomly selected between July 2012 and July 2013. The patients were confirmed as having breast cancer based on pathological features of tissue specimens. None of the patients received preoperative anti-cancer treatment or had malignancies other than breast cancer. Recurrent breast cancer cases were also excluded. Tumor staging was stratified according to the sixth edition of the tumor-node-metastasis (TNM) classification by the International Union Against Cancer. Patient data including age, gender, histologic diagnosis, tumor grade, and clinical stage were documented. Eighty healthy controls were also selected among individuals who visited our hospital for physical checkups and had no clinical evidence of breast cancer or any other overt diseases. All assays were performed to both breast cancer patients and normal controls except immunohistochemistry. We could not obtain the breast tissues of normal controls because the biopsies of normal controls were rarely conducted and none of them agreed to provide their normal breast tissues.

### 3. HLA-G allele assignment

*HLA-G* alleles were analyzed by nucleotide sequence variations at exons 2, 3, 4, and 5. DNA was extracted from ethylenediaminetetraacetic acid (EDTA)-blood samples with the QIAamp DNA Mini Kit (Qiagen; Düsseldorf, Germany). The procedures were automated by using QIAcube (Qiagen). The exon 2, 3, 4 and 5 were amplified using the following primer sequences: G_F (TTCTCTCCTCCTTCTCCTAACC) in the 5′UTR and G_R2 (TCACCCCTTCCTTACCTGAGC) in the intron 5. PCR was performed under the following amplification conditions: 95°C for 5 minutes followed by 32 cycles of 97°C for 20 seconds, 64°C for 45 seconds, and 72°C for 150 seconds, and a final extension at 72°C for 5 minutes. The PCR products were then purified using the Exonuclease I/Shrimp Alkaline Phosphatase mixture provided by Biowithus Inc. (Seoul, Korea), by incubation at 37°C for 50 minutes and at 85°C for 15 minutes. The purified PCR product was directly sequenced with the primers presented in the Table S2 and under the following conditions: 96°C for 30 seconds followed by 25 cycles at 96°C for 10 seconds, 50°C for 5 seconds, and 60°C for 4 minutes, and finalized at 10°C. The sequences were analyzed using BIOWITHUS SBT Analyzer software and compared to the reference sequences recognized by the World Health Organization (WHO) and the international ImMunoGeneTics information system (IMGT), based on exon polymorphisms. For the presence of *HLA-G* 14-bp polymorphism analysis within the 3′UTR of *HLA-G*, the 3′UTR of *HLA-G* was amplified by PCR and detected by electrophoresis on 3% agarose gel. The primers used for the 14-bp polymorphism analysis were GE14HLAG (GTGATGGGCTGTTTAAAGTGTCACC) and RHG4 (GGAAGGAATGCAGTTCAGCATGA) [13]. The deleted and inserted allele generated a 210-bp and a 224-bp PCR fragment, respectively.

### 4. Immunohistochemistry for HLA-G antigens in tissue

Four micrometer-thick sections of the paraffin-embedded breast tissue blocks were cut and mounted on polylysine-coated slides. After deparaffinization, antigen retrieval was processed in 0.01 M Trizma EDTA buffer (pH 9.0) using a microwave oven. Endogenous peroxidase was blocked for 10 minutes in a 3.0% hydrogen peroxide solution at room temperature. Anti-HLA-G mAb (1:50; Enzo Life Sciences International, Inc.; Butler Pike, PA, USA) was added and incubated for one hour at room temperature, and overnight at 4°C. The clone of monoclonal antibody was MEM-G/1 and the antibody was designed to recognize the membrane bound isoform HLA-G (human denatured HLA-G heavy chain). After washing with 0.01 M phosphate-buffered saline (PBS) solution, the binding sites of the primary antibody were visualized through secondary antibody and di-amino benzidine solution using a Dako EnVison kit (Dako; Glostrup, Denmark). Sections were counterstained with hematoxylin, dehydrated, and mounted. All of the slides were stained concurrently to avoid inter-assay variation.

HLA-G immunoreactivity was determined in a binary manner based on the percentage of HLA-G-positive tumor cells. The cut-off value for determining positivity of HLA-G expression was 25% of staining area, irrespective of staining intensity.

### 5. Assay for serum sHLA-G

Serum sHLA-G levels were measured using the Exbio/BioVendor sHLA-G enzyme-linked immunosorbent assay (ELISA) kit (Enzo Life Sciences International, Inc.; Butler Pike, PA, USA). In the assay, calibrators and samples were incubated in microplate wells coated with monoclonal anti-human sHLA-G antibody. After incubation for 1 hour and washing procedures, monoclonal anti-human β2-microglobulin antibody, labeled with horseradish peroxidase (HRP), was added to the wells and incubated for 1 hour. Following the washing process, the bound HRP conjugate reacted with a substrate (tetramethylbenzidine), and the reactions were read as optical densities (ODs) on an automated ELISA plate reader. Finally, sHLA-G concentrations (U/mL) in the samples were calculated using the calibration curve constructed by plotting the ODs against concentrations of calibrators provided by the manufacturer.

### 6. Determination of CA15-3 and CEA

CA15-3 levels were assayed by using the Vitros-3600 system (Ortho-Clinical Diagnostics; Rochester, NY, USA) with Vitros Immunodiagnostic Products CA15-3 (Ortho-Clinical Diagnostics). Tests for CEA were performed using the UniCel DxI 800 Access Immunoassay System with the CEA test kit (Beckman Coulter Inc.; Brea, CA, USA). Each assay was performed according to the respective manufacturer's instructions based on chemiluminescent reactions.

### 7. Statistical analysis

Statistical analyses were performed using Analyse-it Method Evaluation Edition version 2.22 software (Analyse-it Software Ltd.; City West Business Park, Leeds, UK) and PASW version 18.0 (formerly SPSS statistics, SPSS Inc.; Chicago, IL, USA). Multiple comparisons among continuous variables of the study groups were performed using the Kruskal-Wallis test with pairwise comparison and Bonferroni correction to compensate for alpha statistical errors. Continuous variables were also compared between two groups by using Mann-Whitney U tests, and categorical variables of the study groups were assessed using chi-square tests. The correlation between the levels of sHLA-G with CA15-3 and CEA were analyzed by Spearman's rank test. Receiver operating characteristic (ROC) curve analysis was performed to evaluate the diagnostic performances of sHLA-G, CA15-3, and CEA for differentiating breast cancer (n = 80) from healthy control (n = 80) samples. The AU-ROC of sHLA-G for breast cancer detection was compared to those of CA15-3 and CEA. The Pearson chi-square test was used to determine the association between HLA-G expression and clinicopathological parameters in the immunohistochemical studies. Binary logistic regression analysis was performed with breast cancer as the dependent variable and the *HLA-G* genotypes as co-variables to calculate odds ratios (ORs). *P*-values less than 0.05 were considered statistically significant in all analyses.

## Results

### 1. Characteristics of the study groups

No significant difference was observed between the age of the subjects in all groups, according to the TNM stages, except between the control and overall breast cancer groups. The median age for the breast cancer group was 52.0 years (1st to 3rd quartiles  = 47.0 to 62.2 years), while that of the control group was 44.5 years (1st to 3rd quartiles  = 34.0 to 58.6 years). Only female subjects were included in this study.

### 2. The association of HLA-G alleles and genotypes with breast cancer

The distribution and ORs of the *HLA-G* alleles and genotypes by disease status (with allele frequency of 5% or more) are presented in Table 1. We covered the allele frequency more than 5% according to the criteria of a previous report by Ferguson et al. [21]. In the study population, *HLA-G* *01:04:01 and G*01:01:01/*01:04:01 were the most prevalent allele and genotype, respectively. There was no *HLA-G* polymorphism found that was significantly more frequent in patients with breast cancer than in control subjects, and therefore, no polymorphism was associated with the risk for breast cancer. The allele of the presence of *HLA-G* 3′UTR 14-bp sequence was found less frequently in the breast cancer group than in the control group (19.4 versus (vs.) 25.0%).

**Table 1:** Frequencies and odds ratios of *HLA-G* genotypes in breast cancer patients and control subjects.

Genotypes	Control	Breast cancer	P-value^1^	OR	95% CI	P-value^2^
N	80 (%)	80 (%)				
**Allele**						
G*01:01:01	54 (33.8)	59 (36.9)	0.3855	-	-	-
G*01:01:02	12 (7.5)	16 (10.0)	0.4053	1.22	0.53–2.81	0.6400
G*01:01:03	10 (6.3)	7 (4.4)	0.4415	0.64	0.23–1.80	0.3987
G*01:04:01	63 (39.4)	70 (43.8)	0.1395	1.02	0.621.68	0.9477
+14 bp	40 (25.0)	31 (19.4)	0.2260	0.72	0.42–1.23	0.2270
**Genotype**						
G*01:01:01/*01:04:01	20 (25.0)	19 (23.8)	0.8539	-	-	-
G*01:04:01/*01:04:01	13 (16.3)	20 (25.0)	0.1714	1.62	0.63–4.14	0.3143
G*01:01:01/*01:01:01	8 (10.0)	15 (18.8)	0.1147	1.97	0.68–5.72	0.2101
G*01:01:01/*01:01:02	5 (6.3)	5 (6.3)	1.0000	1.05	0.26–4.22	0.9423
G*01:01:03/*01:04:01	5 (6.3)	4 (5.0)	0.7315	0.84	0.20–3.62	0.8172
G*01:01:01/*01:01:03	5 (6.3)	3 (3.8)	0.4682	0.63	0.13–3.02	0.5645
G*01:01:02/*01:04:01	3 (3.8)	5 (6.3)	0.4682	1.75	0.37–8.37	0.4809
−14 bp/−14 bp	44 (55.0)	54 (67.5)		-	-	-
−14 bp/+14 bp	32 (40.0)	21 (26.3)	0.0694	0.53	0.27–1.05	0.0709
+14 bp/+14 bp	4 (5.0)	5 (6.3)	0.9791	1.02	0.26–4.20	0.9791

Abbreviations: N, number of subjects; OR, odds ratio; CI, confidence interval; HLA-G, human leukocyte antigen-G; bp, base pair; −14 bp, absence of *HLA-G* 3′UTR 14-bp sequence; +14 bp, presence of *HLA-G* 3′UTR 14-bp sequence.

Data are shown as numbers of cases and percentages.

1 The P-values were calculated using chi-square tests.

2 The P-values were from binary logistic regression analyses.

### 3. Concentration of sHLA-G according to clinicopathological parameters and HLA-G genotypes

**Figure 1:**
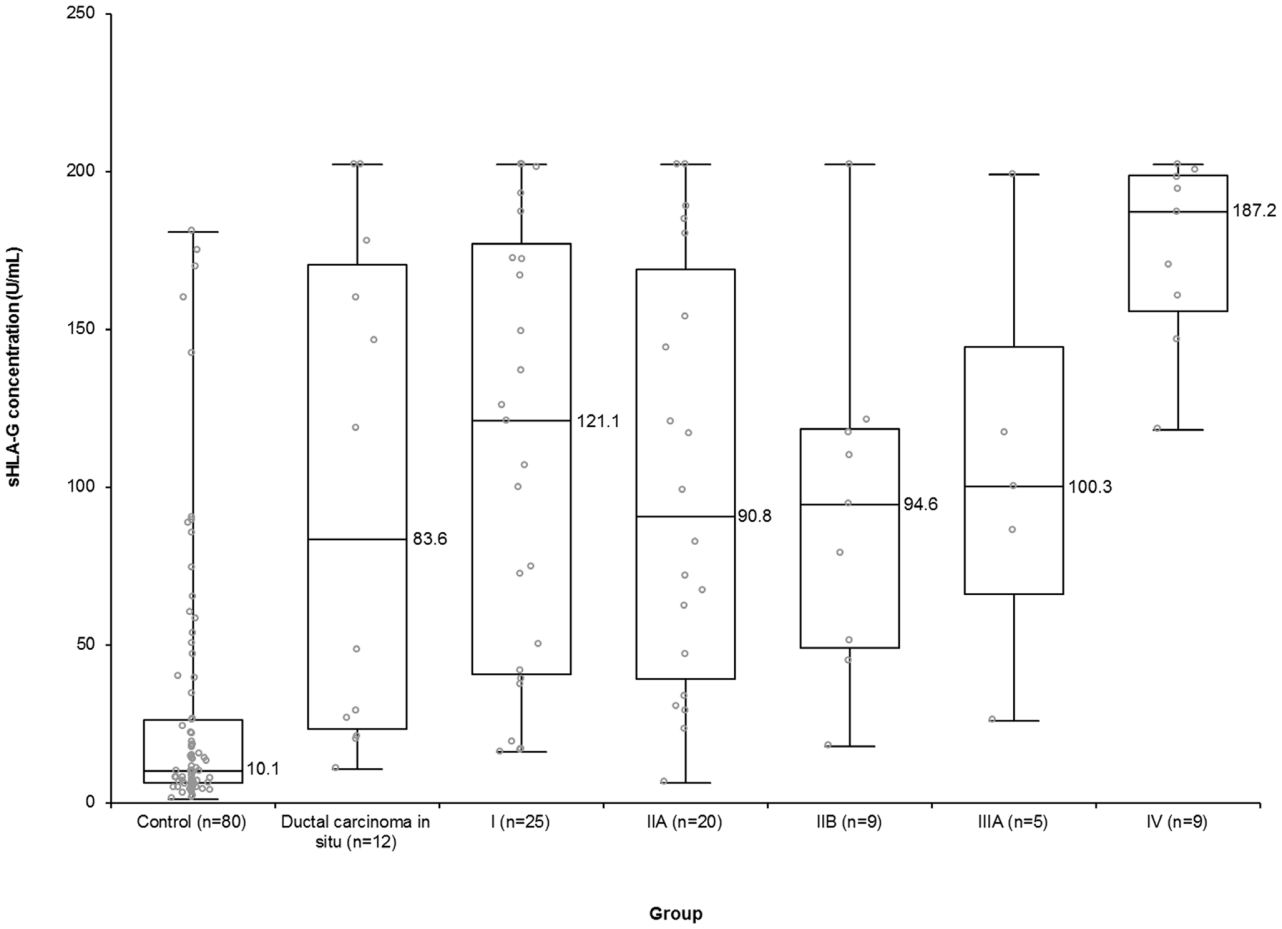
Serum sHLA-G concentrations in the control group and breast cancer group based on TNM stage. Levels of sHLA were significantly increased in patients with breast cancer compared to healthy individuals (*P*<0.001). The median sHLA-G concentration of stage IV patients was significantly higher than that of all other groups (*P*<0.001). The upper and lower ends of boxes and inner lines correspond to the upper and lower quartiles and median values, respectively. The median values of the groups were presented on the right side of the inner lines. Whiskers indicate minimum and maximum values, and circles denote individual values.

The clinicopathological characteristics of the breast cancer group are shown in Table 2. Levels of sHLA-G were significantly associated with metastasis and modified TNM stage (*P*<0.05). Figure 1 also shows the serum concentrations of sHLA-G in the control group and based on the stages of disease in the breast cancer group. Levels of sHLA-G were significantly higher in patients with breast cancer than in the control group (median sHLA-G 117.2 (1st to 3rd quartiles  = 45.9–179.2) U/mL and 10.1 (1st to 3rd quartiles  = 6.3–26.4) U/mL, respectively; *P*<0.001). All breast cancer stage groups had statistically higher sHLA-G values than the control group and all *P*-values were <0.001. In addition, the median sHLA-G level of stage IV was significantly higher than that of all other groups (187.2 U/mL for metastasis and 30.7 U/mL for non-metastasis; *P*<0.001).

**Table 2:** sHLA-G based on clinicopathological parameters, HLA-G expression in tissue, and the *HLA-G* 14-bp sequence status.

Parameters	Value (n, %)	sHLA-G level (U/mL)	P-value^1^
Age	<52^2^ (37, 46.3)	117.3 (60.6–181.0)	0.5021
	≥52 (43,53.8)	99.9 (37.7–178.8)	
TNM stage	0 (12, 15.0)	83.6 (23.5–170.5)	0.1267
	I (25, 31.3)	121.1 (40.9–177.3)	
	IIA (20, 25.0)	90.8 (39.3–169.2)	
	IIB (9, 11.3)	94.6 (49.2–118.6)	
	IIIA (5, 6.3)	100.3 (66.4–144.5)	
	IV (9, 11.3)	187.2 (155.9–198.9)	0.0049^3^
Modified TNM stage^4^	I and II (54, 79.4)	103.3 (44.8–172.2)	0.0336
	III and IV (14, 20.6)	165.5 (115.8–198.3)	
Nuclear grade	1 (4, 6.8)	154.9 (78.6–198.4)	0.4639
	2 (36, 61.0)	103.3 (43.2–161.6)	
	3 (19, 32.2)	79.1 (33.9–166.4)	
Estrogen receptor status	Negative (18, 22.5)	94.8 (30.5–201.5)	0.8627
	Positive (62, 77.5)	117.8 (48.3–178.1)	
Progesterone receptor status	Negative (36, 45.0)	117.9 (38.2–186.2)	0.9614
	Positive (44, 55.0)	117.1 (47.6–170.0)	
HER2 status	Negative (28, 35.0)	102.9 (46.5–134.6)	0.1929
	Positive (52, 65.0)	131.4 (41.4–188.1)	
*HLA-G* allele	−14 bp	120.9 (48.3–187.2)	0.1781
	+14 bp	97.2 (30.5–138.8)	
*HLA-G* genotype	−14 bp/−14 bp	120.9 (48.3–187.2)	0.3922
	−14 bp/+14 bp	106.8 (29.6–144.7)	
	+14 bp/+14 bp	71.8 (30.1–134.0)	
HLA-G in tissue	Negative (41, 56.2)	99.9 (36.2–168.7)	0.4264
	Positive (32, 43.8)	117.8 (54.4–174.7)	

Abbreviations: sHLA-G, soluble human leukocyte antigen-G; HLA-G, human leukocyte antigen-G; HER2, receptor for human epidermal growth factor; bp, base pair; −14 bp, absence of *HLA-G* 3′UTR 14-bp sequence; +14 bp, presence of *HLA-G* 3′UTR 14-bp sequence.

Data are shown as numbers of cases and percentages or ‘median (1st to 3rd quartiles)’.

1 The P-values were calculated using the Mann-Whitney *U* test and Kruskal-Wallis test with Bonferroni correction.

2 The median age of the breast cancer group was 52.0 years.

3 The median sHLA-G level of stage IV patients was significantly higher than those of other stages within the breast cancer group.

4 The modified TNM stage was stratified according to Chen et al. [27]

### 4. Diagnostic performance of serum sHLA-G for breast cancer

**Figure 2:**
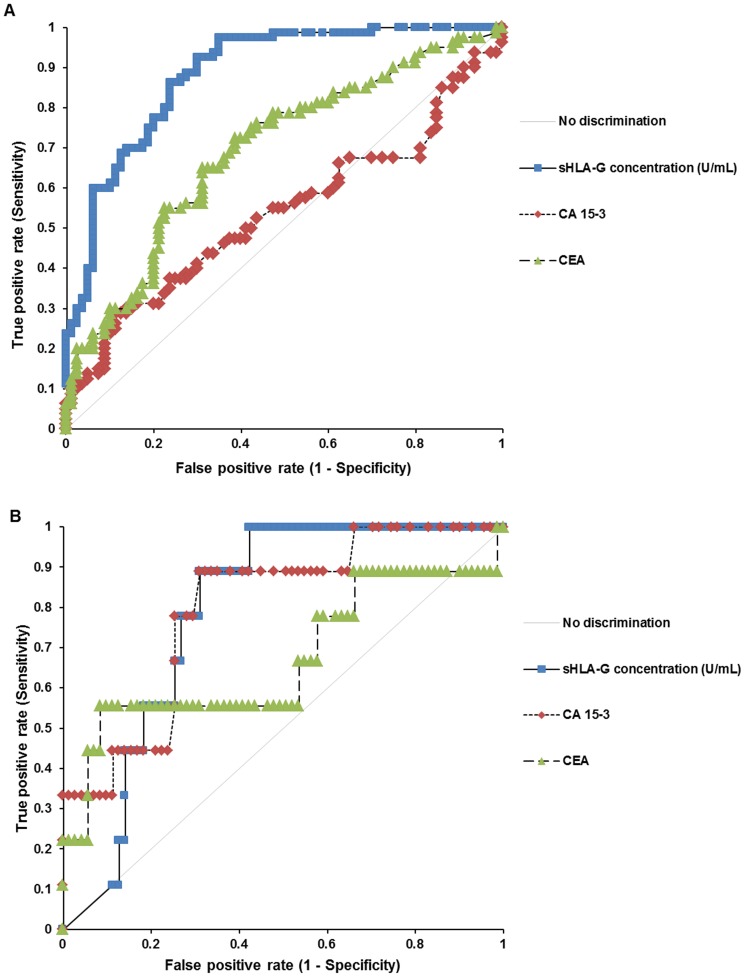
The AU-ROCs of sHLA-G, CA15-3, and CEA for predicting breast cancer. (A) The AU-ROC of sHLA-G for discriminating the breast cancer group (n = 80) from the control group (n = 80) was 0.89 (95% CI = 0.83 to 0.94, *P*<0.001), and was greater than those of CA15-3 (0.54, 95% CI = 0.45 to 0.63, *P* = 0.2116) and CEA (0.69, 95% CI = 0.61 to 0.78, *P*<0.001). (B) The AU-ROCs of sHLA-G, CA15-3, and CEA for differentiating metastasis (n = 9) from other groups (n = 71) were 0.79 (95% CI = 0.68 to 0.90, *P*<0.001), 0.80 (95% CI = 0.65 to 0.95, *P*<0.001), and 0.67 (95% CI = 0.43 to 0.91, *P* = 0.0837), respectively.

Figure 2A illustrates the ROC curves of sHLA-G, CA15-3, and CEA for discriminating patients with breast cancer (n = 80) from the control group (n = 80). There were significant differences among the AU-ROCs of sHLA-G, CA15-3, and CEA (*P*<0.001; sHLA-G vs. CA15-3, *P*<0.001; sHLA-G vs. CEA, *P* = 0.001; CA15-3 vs. CEA, *P* = 0.0106). When the best cut-off was determined by the maximized sum of sensitivity and specificity, the cut-off values to differentiate the breast cancer group from the control group were 19.4 U/mL. The AU-ROC of sHLA-G for detecting metastasis (stage IV) of breast cancer from all other breast cancer groups was 0.79 (95% CI = 0.68–0.90, *P*<0.001) (Figure 2B), and the sum of sensitivity and specificity in this case was maximal with a cut-off sHLA-G level of 146.7 U/mL. The AU-ROCs, sensitivities, and specificities of sHLA-G, CA15-3, and CEA are summarized in Table 3. In addition, the AU-ROC of sHLA-G for discriminating ductal carcinoma *in situ* (stage 0) from healthy individuals was 0.84 (95% CI = 0.74–0.94, *P*<0.001). We also investigate the correlation of sHLA-G with CA15-3 and CEA. The levels of sHLA-G did not significantly correlate with either the CA15-3 or CEA except for the correlation between sHLA-G and CEA (*r* = 0.21, *P* = 0.0075) in the total study population (Table S1). For the correlation of sHLA-G with age, the sHLA-G showed low positive correlations with age only in the total group according to Spearman's rank test. The correlation coefficients of breast cancer group, normal controls, and total group were 0.01 (95% CI = −0.21 to 0.23, *P* = 0.9358), 0.03 (95% CI = −0.19 to 0.25, *P* = 0.7766), and 0.22 (95% CI = 0.07 to 0.36, *P* = 0.0048), respectively. According to the results of multivariate analysis which control potential confounding factors, both age and sHLA-G were found to be independently relevant to breast cancer (OR = 1.05, 95% CI = 1.02–1.09, *P* = 0.003 for age; OR = 1.03, 95% CI = 1.02–1.03, *P*<0.001 for sHLA-G). Although, slightly affected by age, sHLA-G is still thought to have diagnostic ability for the detection of breast cancer.

**Table 3:** The AU-ROCs, sensitivities, and specificities of sHLA-G, CA15-3, and CEA.

Marker	Breast cancer (n = 80) vs. healthy subjects (n = 80)				Metastasis (n = 9) vs. other groups (n = 71)			
	ROC-AUC^1^	*P* value	Sensitivity (%)^1^	Specificity (%)^1^	ROC-AUC^1^	*P* value	Sensitivity (%)^1^	Specificity (%)^1^
sHLA-G	0.89 (0.83 to 0.94)	<0.001	92.5 (84.4 to 97.2)	70.0 (58.7 to 79.7)	0.79 (0.68 to 0.90)	<0.001	88.9 (51.8 to 99.7)	69.0 (56.9 to 79.5)
CA 15-3	0.54 (0.45 to 0.63)	0.2116	28.8 (19.2 to 40.0)	88.8 (79.7 to 94.7)	0.80 (0.65 to 0.95)	<0.001	88.9 (51.8 to 99.7)	69.0 (56.9 to 79.5)
CEA	0.69 (0.61 to 0.78)	<0.001	65.0 (53.5 to 75.3)	68.8 (57.4 to 78.7)	0.67 (0.43 to 0.91)	0.0837	55.6 (21.2 to 86.3)	91.5 (82.5 to 96.8)

Abbreviations: AU-ROCs, areas under the receiver operating characteristic curves; sHLA-G, soluble human leukocyte antigen-G; CEA, carcinoembryonic antigen.

1 Shown as ‘values (95% CI)’.

### 5. HLA-G expression in tissue related to clinicopathological parameters and HLA-G genotypes

**Figure 3:**
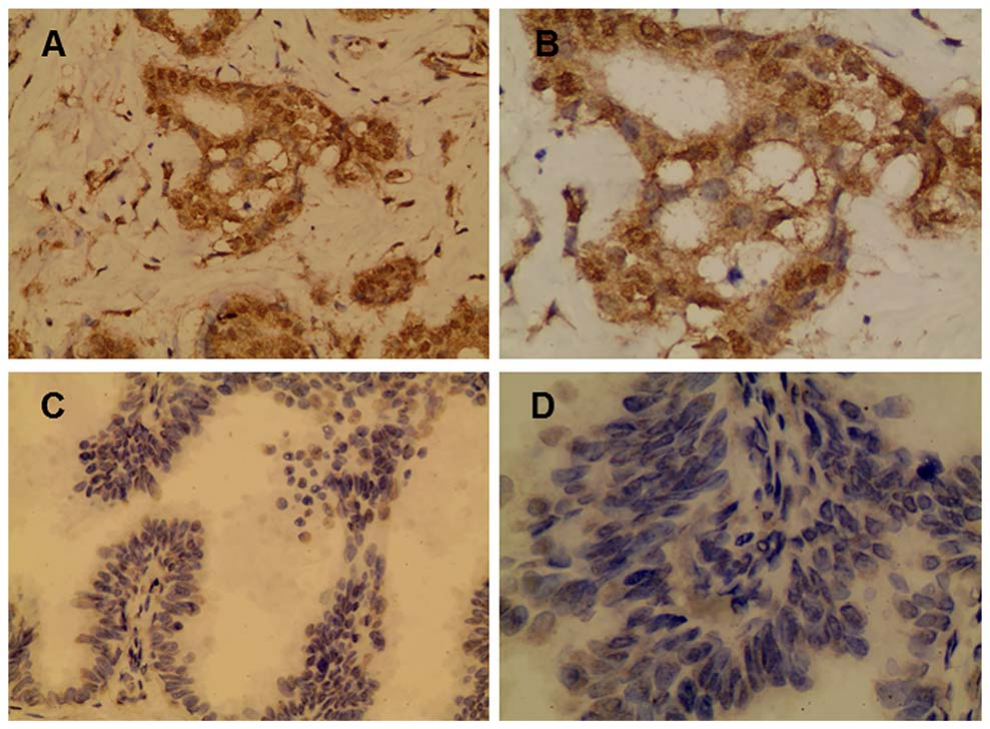
Immunohistochemistry staining of HLA-G expression in breast cancer lesions. Positive HLA-G staining (original magnification, ×200 for A and ×400 for B). Negative HLA-G staining (original magnification, ×200 for C and ×400 for D).

HLA-G expression was visualized by immunohistochemistry as a brown-stained product (Figure 3). HLA-G was observed in 43.8% (32/73) of breast cancer lesions. HLA-G was expressed in 18.8% (3/16) of patients with estrogen receptor (ER)-negative breast cancer and 50.9% (29/57) of patients with ER-positive breast cancer. Furthermore, HLA-G expression in breast cancer lesions was significantly associated with the homozygous presence of 14-bp, with a *P*-value of 0.0407. However, HLA-G expression was not significantly associated with other parameters, such as age and disease stages (Table 4).

**Table 4:** Association of HLA-G expression in cancer lesions with clinicopathological parameters, and *HLA-G* 14-bp sequence status.

Parameter	Value	No. of cases	HLA-G expression (n)		P-value^1^
			Negative (%)	Positive (%)	
Age (years)	<52^2^	35	17 (48.6)	18 (51.4)	0.2096
	≥52	38	24 (63.2)	14 (36.8)	
TNM stage	0	12	9 (75.0)	3 (25.0)	0.2640
	I	25	16 (64.0)	9 (36.0)	
	IIA	20	9 (45.0)	11 (55.0)	
	IIB	9	5 (55.6)	4 (44.4)	
	IIIA	5	2 (40.0)	3 (60.0)	
	IV	2	0 (0.0)	2 (100.0)	
Modified TNM stage^3^	I and II	54	30 (55.6)	24 (44.4)	0.1786
	III and IV	7	2 (28.6)	5 (71.4)	
Nuclear grade	1	4	2 (50.0)	2 (50.0)	0.9221
	2	36	19 (52.8)	17 (47.2)	
	3	19	11 (57.9)	8 (42.1)	
Estrogen receptor status	Negative	16	13 (81.3)	3 (18.8)	0.0221
	Positive	57	28 (49.1)	29 (50.9)	
Progesterone receptor status	Negative	31	20 (64.5)	11 (35.5)	0.2166
	Positive	42	21 (50.0)	21 (50.0)	
HER2 status	Negative	26	11 (42.3)	15 (57.7)	0.0760
	Positive	47	30 (63.8)	17 (36.2)	
HLA allele	G*01:01:01	56	19 (33.9)	37 (66.1)	0.8504
	G*01:01:02	15	5 (33.3)	10 (66.7)	
	G*01:01:03	7	2 (28.6)	5 (71.4)	
	G*01:04:01	60	16 (26.7)	44 (73.3)	
	−14 bp	47	24 (51.1)	23 (48.9)	0.2377
	+14 bp	26	17 (65.4)	9 (34.6)	
HLA genotype	G*01:01:01/*01:04:01	18	5 (27.8)	13 (72.2)	0.2441
	G*01:04:01/*01:04:01	16	4 (25.0)	12 (75.0)	
	G*01:01:01/*01:01:01	14	6 (42.9)	8 (57.1)	
	G*01:01:01/*01:01:02	5	0 (0.0)	5 (100.0)	
	G*01:01:03/*01:04:01	4	0 (0.0)	4 (100.0)	
	G*01:01:01/*01:01:03	3	2 (66.7)	1 (33.3)	
	G*01:01:02/*01:04:01	4	2 (50.0)	2 (50.0)	
	−14 bp/−14 bp	47	24 (51.1)	23 (48.9)	0.1104
	−14 bp/+14 bp	21	12 (57.1)	9 (42.9)	
	+14 bp/+14 bp	5	5 (100.0)	0 (0.0)	0.0407

Abbreviations: HLA, human leukocyte antigen-G; HER2, a receptor for human epidermal growth factor; −14 bp, absence of *HLA-G* 3′UTR 14-bp sequence; +14 bp, presence of *HLA-G* 3′UTR 14-bp sequence.

Data are shown as numbers of cases and percentages.

1 Comparison of HLA-G expression status between each parameter using the Pearson chi-square test.

2 The median age of the breast cancer group was 52.0 years.

3 The modified TNM stage was stratified according to Chen et al. [27].

## Discussion

The potential clinical relevance of HLA-G in cancer was previously addressed in several studies [4], [5]. The HLA-G system plays a critical role in the recognition of tumor antigens and the relevant immune responses against carcinoma. The *HLA-G* gene might be responsible for the inhibition of T and NK cells, facilitating tumor escape from immune surveillance [21], [22]. Furthermore, both membrane-bound and sHLA-G isoforms perform inhibitory functions through binding to specific receptors of immune regulatory cells [9], [10], [27], [28]. However, the definite mechanism of action of HLA-G in breast cancer remains unknown. A goal of this study was to estimate the potential role of HLA-G in breast carcinogenesis through investigating *HLA-G* polymorphisms and expression in both the tissue and serum in the same patient population.

In terms of the clinical impact of *HLA-G* on cancer, the G*01:01:02 allele, which was observed in 7.5% of the control group and 10.0% of the breast cancer group in our study, showed significantly increased risk for invasive cervical cancer (OR = 3.52, 95% CI = 1.43–8.61, *P* = 0.006 for homozygote) [21]. However, in the present study, homozygotes of G*01:01:02 were not found because of limited allelic polymorphism in the Korean population. However, heterozygous genotypes for G*01:01:02, such as G*01:01:01/*01:01:02 and G*01:01:02/*01:04:01, were observed in our study, and were not strongly associated with the risk of breast cancer. These results are similar to previously reported results on the association between G*01:01:02 heterozygote genotype and cervical cancer (OR = 0.92, 95% CI = 0.50–1.68) [21].

The 3′UTR 14-bp polymorphism was reported to be related to the magnitude of HLA-G production by modulating HLA-G mRNA stability [29], [30]. Although the mechanisms have not been clearly elucidated, *HLA-G* alleles with the presence of 14-bp sequence (5′-ATTTGTTCATGCCT-3′) have been associated with low HLA-G mRNA production [29]. HLA-G mRNA transcripts with the presence of 14-bp could be further processed by the removal of the first 92 bases of exon 8, yielding smaller HLA-G transcripts known to be more stable than the complete mRNA forms [29], [30]. Regarding sHLA-G, the heterozygous and homozygous genotypes for the presence of 14-bp (−14 bp/+14 bp and +14 bp/+14 bp) had lower sHLA-G concentrations compared to the homozygote form of the absence of 14-bp (−14 bp/−14 bp), both in the breast cancer group and among all subjects including the control group. Although these observations were not statistically significant, Chen et al. [27] documented that the sHLA-G level was significantly lower in carriers of the +14 bp/+14 bp genotype than in those with −14 bp/+14 bp (*P* = 0.004) and −14 bp/−14 bp genotypes (*P* = 0.003). Thus, the influence of the presence of *HLA-G* 14-bp polymorphism on sHLA-G expression and its potential biological functions should be further investigated and interpreted with caution.

Regarding the expression of HLA-G in breast tissue, several studies showed that 41–66% of breast cancer lesions expressed HLA-G [5]. In the present study, HLA-G was expressed in 43.8% (32/73) of breast cancer tissues. In addition, He et al. [26] demonstrated that HLA-G expression was significantly associated with the ER status, which was concordant with our data. These results might be related to the effects of TAM and RU486, which function as anti-breast cancer drugs through blocking ER receptors [31]. Furthermore, the homozygous genotype for the presence of 14-bp was related to HLA-G expression, indicating that HLA-G status might be involved in breast carcinogenesis.

There were several methods for the evaluation of staining, which could affect the results of HLA-G expression in tissues. He et al. [26] graded HLA-G expression as follows: (−) for tissue specimens without staining, (+) for tissue specimens with less than 25% of cancer tissue and/or weakly stained, (++) for tissue specimens with 25–50% of cancer tissue and/or moderately stained, and (+++) for tissue specimens with >50% of cancer tissue and/or strongly stained. Chen et al. [27] divided staining results into four categories: negative, 1 for 1–25%, 2 for 26–50%, 3 for 51–75%, and 4 for >75%. Kruijf et al. [32] scored HLA-G in a binary manner, considering any specific staining of tumor cells as positive expression and no staining as no expression. We followed the all of the three previous criteria and analyzed our data preliminarily. However, the numbers of patients in the subdivided groups were too small to conclude statistically significant results with the four grade system. Therefore, we selected the binary manner with the cut-off at 25% of staining area, which distinguish between weakly and moderately stained, and found the relations between HLA-G with breast cancer.

sHLA-G levels in the sera from patients with breast cancer and healthy individuals were also investigated. sHLA-G concentrations in the breast cancer group were significantly elevated compared to those in the control group, which is similar to previously published data. Chen et al. [27] reported that plasma sHLA-G in breast cancer patients was significantly higher than that of control subjects (median, 82.19 U/mL vs. 9.65 U/mL, *P*<0.001). In particular, sHLA-G levels were significantly increased even in patients with ductal carcinoma *in situ* (stage 0) who might have to undergo breast-conserving surgery and radiation, in comparison to healthy controls (*P*<0.001) [33]. Our findings suggest that sHLA-G could already be up-regulated in precancerous conditions.

To date, very few studies have been published regarding the clinical implications of sHLA-G in breast cancer in relation to clinicopathological characteristics. Circulating sHLA-G levels are not significantly correlated to most clinicopathological parameters, such as the age of the patients, location, TNM stage, tumor stage, and nuclear grade, according to previous data [26], [27], [34]. Similarly, we also found no correlation between sHLA-G and the clinicopathological parameters of our sample population. However, the median sHLA-G level of stage IV patients was significantly higher than those of patients in other stages both within the breast cancer group (*P* = 0.0049) and within the entire population analyzed (*P*<0.001) in this study. These findings suggest that circulating sHLA-G could have the potential to differentiate breast cancer with metastasis from breast cancer without metastasis, which was not previously discussed in the literature. Furthermore, sHLA-G showed an association with modified TNM stage, which combined stage I with II and stage III with IV according to Chen et al. [27]. Combining these stages increased the sample size, and therefore the power to obtain significant *P*-values.

The ROC curve analyses were performed to evaluate the feasibility of sHLA-G as a diagnostic marker for breast cancer. Previous research showed that the AU-ROC of sHLA-G in discriminating between breast cancer patients and healthy controls was 0.735 (95% CI = 0.630–0.874, *P*<0.001) [7]. They measured not serum sHLA-G, but plasma sHLA-G in the 120 patients with breast cancer and 40 healthy controls. The breast cancer group was composed of 40 invasive ductal carcinoma, 40 ductal carcinoma *in situ* and 40 lobular neoplasia. In another study, the value of AU-ROC was 0.953 (95% CI = 0.926–0.981, *P*<0.001) from breast cancer patients vs. normal controls [27]. They also determined the plasma sHLA-G in 92 primary ductal breast cancer patients and in 70 normal controls. In our study, the AU-ROCs for distinguishing breast cancer from control subjects was 0.89, which is intermediate between the two previous results and was higher than those obtained for CA15-3 and CEA. The variations of the AU-ROCs among these studies could be caused by the differences of population set and study size. Further, there was no information regarding the comparison of sHLA-G with CA15-3 and CEA in breast cancer, to the best of our knowledge. In addition, the AU-ROC of sHLA-G for discriminating ductal carcinoma *in situ* from normal specimens was 0.84, showing the prominent diagnostic utility of sHLA-G.

The AU-ROC of sHLA-G for differentiating breast cancer metastasis from other stages of breast cancer was 0.79, which have not been previously documented. Barrier et al. [35] evaluated the ability of HLA-G to discriminate between metastatic and non-metastatic cancer using immunohistochemistry in the 44 cases of endometrial adenocarcinoma. The AU-ROC value was 0.754 (95% CI = 0.590–0.916) for metastatic vs. non-metastatic cancer and the specificity to predict metastatic disease was 86% (95% CI = 0.68–0.95), while the sensitivity was 63% (95% CI = 0.39–0.87). They concluded that HLA-G may serve as a clinical marker for the preoperative prediction of metastatic endometrial cancer. We followed the criteria of the Barrier et al. [35] to estimate the ability of sHLA-G for differentiating the metastatic and non-metastatic breast cancer. Although, the AU-ROC value of CA15-3 for differentiating breast cancer metastasis from other stages of breast cancer (0.80) was slightly higher than that of sHLA-G (0.79), the AU-ROC of sHLA-G for discriminating the breast cancer group from normal controls (0.89) was prominently higher than that of CA15-3 (0.54). Thus, serum sHLA-G concentration may potentially be a diagnostic marker for breast cancer and metastasis.

In this study, the limited allelic polymorphism of *HLA-G* in the Korean population is one of the inevitable limitations for analyzing genotype associations. Further studies with a large number of specimens would facilitate determining the clinical usefulness of HLA-G polymorphism and sHLA-G as a diagnostic marker.

## Conclusions

HLA-G seems to be implicated in the immune escape mechanisms of breast cancer. The presence of 14-bp of *HLA-G* was associated with breast cancer susceptibility according to the results of HLA-G expression in tissue. The circulating sHLA-G levels were markedly increased in patients with breast cancer, including ductal carcinoma *in situ*. In addition, the AU-ROCs of sHLA-G for differentiating metastasis from all other groups were high enough to determine the presence of breast cancer metastasis. Therefore, the 14-bp *HLA-G* gene polymorphism could be involved in breast carcinogenesis, and the measurement of sHLA-G concentrations have diagnostic value for detecting breast cancer and metastasis.

## Supporting Information

Table S1 **Correlation between sHLA-G with CA15-3 and CEA.** The levels of sHLA-G did not significantly correlate with either the CA15-3 or CEA except for the correlation between sHLA-G and CEA (*r* = 0.21, *P* = 0.0075) in the total study population. The correlation between the sHLA-G with CA15-3 and CEA were analyzed by Spearman's rank test.
(DOC)

Table S2 **Nucleotide sequences of the primers used in direct sequencing of HLA-G.** The primers for direct sequencing of HLA-G were presented with their positions and names.
(DOC)
