# Supplementary material for: Human Leukocyte Antigen-G (HLA-G) Polymorphism and Expression in Breast Cancer Patients
Source: PLoS One. 2014 May 28;9(5):e98284. doi: 10.1371/journal.pone.0098284 (PMC4037222; doi:10.1371/journal.pone.0098284)
Supplement: Table S1 — Correlation between sHLA-G with CA15-3 and CEA. The levels of sHLA-G did not significantly correlate with either the CA15-3 or CEA except for the correlation between sHLA-G and CEA (r = 0.21, P = 0.0075) in the total study population. The correlation between the sHLA-G with CA15-3 and CEA were analyzed by Spearman's rank test. (DOC) [file pone.0098284.s001.doc]

| Table S1. Correlation between sHLA-G with CA15-3 and CEA | | | |  |
| --- | --- | --- | --- | --- |
| Group (n) | Parameters | sHLA-G | | |
|  |  | *r* | 95% CI | P value |
| Breast cancer (80) | CA15-3 | -0.03 | -0.25 to 0.19 | 0.7909 |
|  | CEA | 0.01 | -0.21 to 0.23 | 0.9089 |
| Control (80) | CA15-3 | -0.17 | -0.38 to 0.05 | 0.1225 |
|  | CEA | 0 | -0.22 to 0.22 | 0.9677 |
| Total (160) | CA15-3 | -0.02 | -0.17 to 0.14 | 0.8048 |
|  | CEA | 0.21 | 0.06 to 0.35 | 0.0075 |
| Abbreviations: CI, confidence interval. | | |  |  |
