# Supplementary material for: Human Leukocyte Antigen-G (HLA-G) Polymorphism and Expression in Breast Cancer Patients
Source: PLoS One. 2014 May 28;9(5):e98284. doi: 10.1371/journal.pone.0098284 (PMC4037222; doi:10.1371/journal.pone.0098284)
Supplement: Table S2 — Nucleotide sequences of the primers used in direct sequencing of HLA-G. The primers for direct sequencing of HLA-G were presented with their positions and names. (DOC) [file pone.0098284.s002.doc]

| Table S2. Nucleotide sequences of the primers used in direct sequencing of HLA-G | | | |
| --- | --- | --- | --- |
| Position | Name | 5' Forward 3' | 5' Reverse 3' |
| Exon 2 | G2 | AGTGCGGGGTCAGGA | GGTCGTGATCTGCKC |
| Exon 3 | G3 | GCCTTTACCAAAATC | CATTTTCCTCCTCTCCTTG |
| Exon 4-5 | G4 | GTCTGGGTTCTGTGC | TCCGCAGGGTAGAAGC |
